# Tools for genetic engineering and gene expression control in *Novosphingobium aromaticivorans* and *Rhodobacter sphaeroides*

**DOI:** 10.1128/aem.00348-24

**Published:** 2024-09-26

**Authors:** Ashley N. Hall, Benjamin W. Hall, Kyle J. Kinney, Gabby G. Olsen, Amy B. Banta, Daniel R. Noguera, Timothy J. Donohue, Jason M. Peters

**Affiliations:** 1 DOE Great Lakes Bioenergy Research Center, University of Wisconsin-Madison, Madison, Wisconsin, USA; 2 Pharmaceutical Sciences Division, School of Pharmacy, University of Wisconsin-Madison, Madison, Wisconsin, USA; 3 Laboratory of Genetics, University of Wisconsin-Madison, Madison, Wisconsin, USA; 4 Department of Civil and Environmental Engineering, University of Wisconsin-Madison, Madison, Wisconsin, USA; 5 Department of Bacteriology, University of Wisconsin-Madison, Madison, Wisconsin, USA; 6 Department of Medical Microbiology and Immunology, University of Wisconsin-Madison, Madison, Wisconsin, USA; 7 Center for Genomic Science Innovation, University of Wisconsin-Madison, Madison, Wisconsin, USA; Washington University in St. Louis, St. Louis, Missouri, USA

**Keywords:** synthetic biology, CRISPR-Cas, Mobile-CRISPRi, Alphaproteobacteria, bioproducts, genome engineering

## Abstract

Alphaproteobacteria have a variety of cellular and metabolic features that provide important insights into biological systems and enable biotechnologies. For example, some species are capable of converting plant biomass into valuable biofuels and bioproducts that have the potential to contribute to the sustainable bioeconomy. Among the Alphaproteobacteria, *Novosphingobium aromaticivorans*, *Rhodobacter sphaeroides*, and *Zymomonas mobilis* show promise as organisms that can be engineered to convert extracted plant lignin or sugars into bioproducts and biofuels. Genetic manipulation of these bacteria is needed to introduce engineered pathways and modulate expression of native genes with the goal of enhancing bioproduct output. Although recent work has expanded the genetic toolkit for *Z. mobilis*, *N. aromaticivorans* and *R. sphaeroides* still need facile, reliable approaches to deliver genetic payloads to the genome and to control gene expression. Here, we expand the platform of genetic tools for *N. aromaticivorans* and *R. sphaeroides* to address these issues. We demonstrate that Tn*7* transposition is an effective approach for introducing engineered DNA into the chromosome of *N. aromaticivorans* and *R. sphaeroides*. We screen a synthetic promoter library to identify isopropyl β-D-1-thiogalactopyranoside-inducible promoters with regulated activity in both organisms (up to ~15-fold induction in *N. aromaticivorans* and ~5-fold induction in *R. sphaeroides*). Combining Tn*7* integration with promoters from our library, we establish CRISPR (Clustered Regularly Interspaced Short Palindromic Repeats) interference systems for *N. aromaticivorans* and *R. sphaeroides* (up to ~10-fold knockdown in *N. aromaticivorans* and *R. sphaeroides*) that can target essential genes and modulate engineered pathways. We anticipate that these systems will greatly facilitate both genetic engineering and gene function discovery efforts in these species and other Alphaproteobacteria.

**IMPORTANCE:**

It is important to increase our understanding of the microbial world to improve health, agriculture, the environment, and biotechnology. For example, building a sustainable bioeconomy depends on the efficient conversion of plant material to valuable biofuels and bioproducts by microbes. One limitation in this conversion process is that microbes with otherwise promising properties for conversion are challenging to genetically engineer. Here we report genetic tools for *Novosphingobium aromaticivorans* and *Rhodobacter sphaeroides* that add to the burgeoning set of tools available for genome engineering and gene expression in Alphaproteobacteria. Our approaches allow straightforward insertion of engineered pathways into the *N. aromaticivorans* or *R. sphaeroides* genome and control of gene expression by inducing genes with synthetic promoters or repressing genes using CRISPR interference. These tools can be used in future work to gain additional insight into these and other Alphaproteobacteria and to aid in optimizing yield of biofuels and bioproducts.

## INTRODUCTION

A myriad of microbial activities has and can have positive impacts on the health of the planet, its inhabitants, and the production of compounds needed by a growing population. In recent years, a diverse group of microbes have been identified that can convert renewable plant material into sustainable biofuels and bioproducts. However, additional genetic tools for these organisms are needed to dissect their metabolic and regulatory networks and to build economically feasible production strains. To generate the knowledge needed to power the future bioeconomy, we need the ability to easily engineer the genomes of additional microbial chassis and to rationally control the expression of bioproduct-relevant genes.

Many Alphaproteobacteria have unique metabolic pathways that make them promising chassis for production of biofuels and bioproducts. *Novosphingobium aromaticivorans* and *Rhodobacter sphaeroides* are two Alphaproteobacterial species of interest for their potential to convert plant feedstocks into bioproducts. *N. aromaticivorans* utilizes aromatic carbon sources and has been engineered to produce bioproducts including carotenoids, pimelic acid, *cis*,*cis*-muconic acid, and 2-pyrone-4,6-dicarboxylic acid, a nylon precursor from plant phenolics (1–4). *R. sphaeroides* is a well-studied photosynthetic bacterium with the capacity to produce biotechnologically useful products such as hydrogen, terpenes, ubiquinone, and polyhydroxyalkanoates (5–9). The genetic tools to manipulate these bacteria include homology-based genome integration, Tn-seq, and a few inducible plasmid vectors (10–13). While effective in specific use cases, additional genetic tools are needed to overcome limitations in homology-based integration, make it easier to target essential or other specific genes, or bypass the use of plasmids.

The ability to generate and control expression of engineered pathways can also be critical for optimizing or altering bioproduct yields. Genomic integration of engineered pathways allows for stable maintenance of the pathway without continuous application of selective pressure (e.g., antibiotics) that is typically required to maintain plasmids (14). Various approaches exist for genomic integration of engineered DNA including homologous recombination, phage integrase systems, and transposons (15–17). Homologous recombination can target DNA to a specific locus. However, single crossovers are unstable without selection, and double-crossovers typically require the use of time-consuming counter-selection processes, making homologous recombination inefficient and low throughput. Integrases require specific sites on the genome to insert their DNA payload. Recent work has shown that arrays of integrase sites can be delivered to recipient genomes and utilized for multiple insertions of cargo (18), although such systems require a two-step procedure that adds the initial integrase sites. Transposon-based integration of DNA has been used in diverse bacteria and is a highly effective, one-step procedure that can be implemented at the library scale. Among transposons, Tn*7* has shown particular value for strain engineering due to its ability to site-specifically integrate downstream of a recognition site in the 3′ end of the *glmS* gene (19) that is broadly conserved in bacteria (20, 21). As such, Tn*7* has become the integration tool of choice in diverse bacteria including the Alphaproteobacterium *Zymomonas mobilis* (19, 22, 23); however, Tn*7* function has neither been demonstrated for *N. aromaticivorans* nor optimized for *R. sphaeroides* (24).

Methods to control gene expression, including inducible promoters and CRISPR interference (CRISPRi) knockdowns, are useful for modulating the levels and temporal dynamics of gene products. Most inducible promoters rely on DNA sequences recognized by *Escherichia coli* σ^70^; however, these elements have received only limited characterization in high-GC Alphaproteobacteria, such as *N. aromaticivorans* (63% GC) and *R. sphaeroides* (68% GC). For instance, the wild-type *lac* promoter from *E. coli* is reported to function poorly in *R. sphaeroides* (25). While inducible promoters have not been systematically characterized in *N. aromaticivorans* to our knowledge, promoters inducible by light, oxygen, crystal violet, and isopropyl β-D-1-thiogalactopyranoside (IPTG) have been tested in *R. sphaeroides* (13, 25–28). Although these foreign promoters can respond to an array of possible inducers, some inducers inhibit *R. sphaeroides* growth (crystal violet) (25) or substantially alter physiological processes of interest (light and oxygen). An IPTG-inducible derivative of the *R. sphaeroides* 16S rRNA promoter has been engineered (9, 25), but it is unclear if the engineered variant is regulated by nutrient availability and cellular factors (e.g., CarD, ppGpp, and DksA) as is the parent promoter (29–32). Synthetic, IPTG-inducible promoters have a potential advantage of not being inherently regulated by cellular systems and of utilizing an inducer that is likely less physiologically disruptive to the host. For example, Ind et al. (13) showed that the synthetic, IPTG-inducible promoter P_A1/04/03_ (33) had excellent induction properties in *R. sphaeroides* when used to regulate expression of a chemotaxis protein (13). Additional synthetic promoters could allow for distinct levels of induction or reduce part redundancy that can destabilize genetic circuits (34). We note that inducible promoters have been developed for other high-GC Alphaproteobacteria, such as *Methylobacterium extorquens* AM1 (35).

The development of facile genetic engineering tools, such as CRISPRi, depends on reliable strategies for integration of CRISPR systems into genomic DNA and inducible expression of CRISPRi components. We previously developed a platform called “Mobile-CRISPRi” that takes advantage of Tn*7*-based integration and synthetic promoters to deliver and induce CRISPRi knockdown in a number of diverse bacteria (22). Developing an effective Mobile-CRISPRi system for *Z. mobilis* required optimization of CRISPRi component expression [single guide RNAS (sgRNAs) and dCas9], a phenomenon we observed in some, but not all, recipient bacteria (22, 23).

In this work, we demonstrate the use of Tn*7* for stable, site-specific integration in *R. sphaeroides* and *N. aromaticivorans*, optimizing transconjugant recovery using several mating schemes. We create a series of synthetic, IPTG-inducible promoters that function in both *N. aromaticivorans and R. sphaeroides*, providing a reliable system for controlled gene expression. Finally, we combine these elements in proof-of-principle experiments to demonstrate effective CRISPRi control systems that can phenotype essential genes in both organisms and control expression of engineered pathways for synthesis of foreign carotenoids in *N. aromaticivorans*.

## RESULTS

### Tn*7* integrates engineered DNA into *N. aromaticivorans* and *R. sphaeroides*

**Fig 1:**
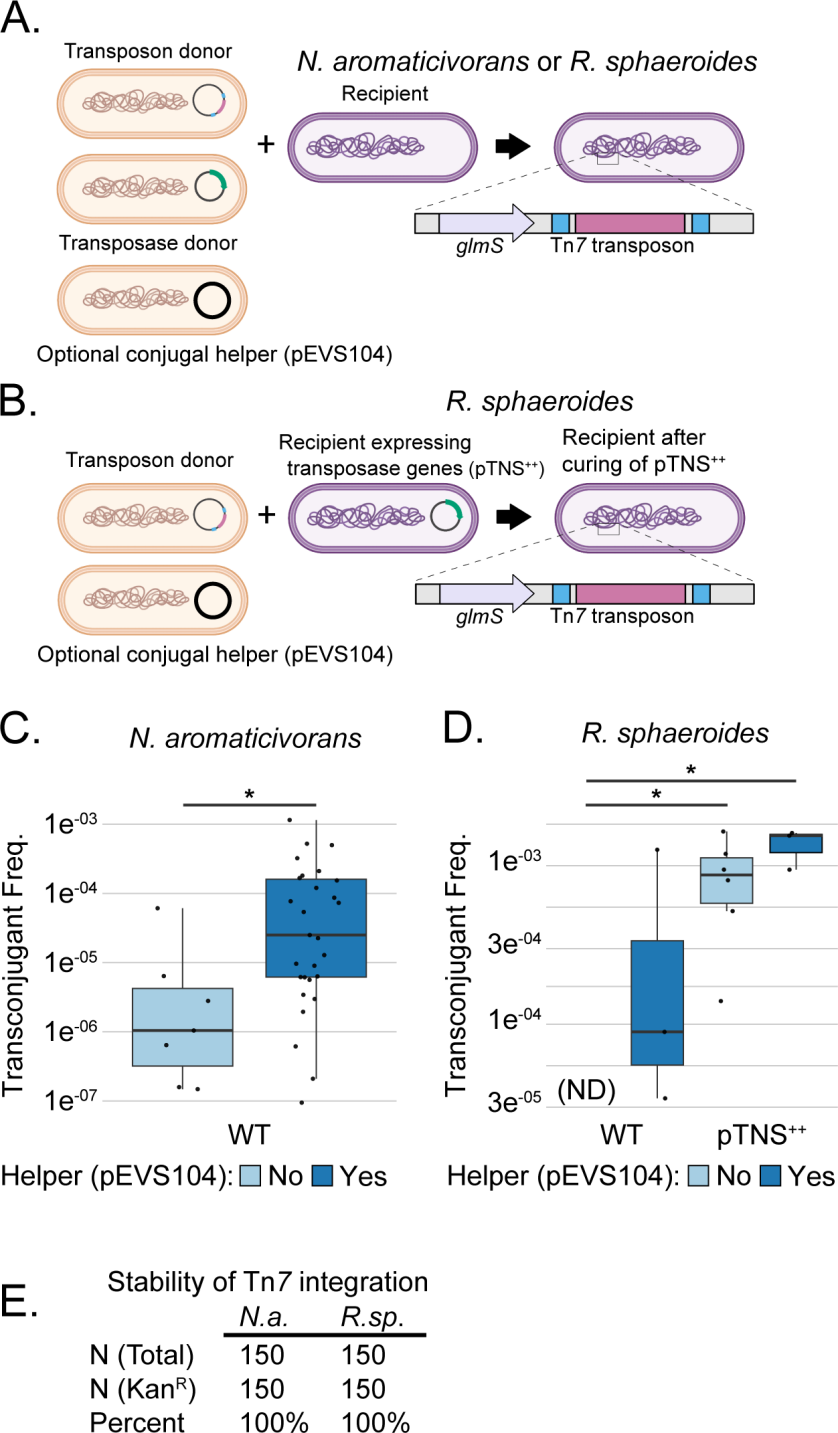
Insertion of Tn*7* into the genomes of *N. aromaticivorans* and *R. sphaeroides*. (**A**) Schematic of a mating scheme for transfer of the Tn*7* transposon and Tn*7* transposase-expressing plasmid into the recipient of interest. (**B**) Schematic of a mating scheme for delivery of a Tn*7* transposon into *R. sphaeroides* 2.4.1 with Tn*7* transposase genes constitutively expressed from a replicative plasmid (*tnsABCD* in pTNS^++^). Growing cells without selection for the plasmid is required to cure pTNS^++^ from recipient cells. (**C and D**) Frequency of Tn*7* transconjugant recovery (Kan^R^/total CFUs) in *N. aromaticivorans* and *R. sphaeroides*, respectively, using different mating schemes. The helper plasmid contains a copy of the RP4 transfer machinery (pEVS104). Points are individual matings. Asterisks indicate statistical significance (*P* < 0.05, *t*-test). (**E**) Stability of a Kan^R^ Tn*7* insertion in both *N. aromaticivorans* and *R. sphaeroides* following serial passaging (~50 generations) in the absence of selection. The *R. sphaeroides* strains used in the stability experiment were not generated using pTNS^++^ and therefore do not contain pTNS^++^. “N” indicates the number of colonies patched on both non-selective (total) and selective (Kan^R^) plates. Related summary statistics and growth curve data can be found in Tables S1 to S3. *N.a.*, *N. aromaticivorans*; ND, no colonies were detected across three replicates; *R.sp*., *R. sphaeroides*.

Site-specific Tn*7* transposition into the Tn*7 att* site downstream of *glmS* is a common method to integrate engineered DNA into the genomes of diverse bacteria (19, 21). We previously used a tri-parental mating scheme to introduce Tn*7* into the genomes of various Gammaproteobacteria (22). In this scheme, a recipient strain was mated with two *E. coli* donors harboring Tn*7* plasmids: one containing Tn*7* transposase genes and a second containing the Tn*7* transposon (22, 23, 36). Donor *E. coli* cells express the RP4 conjugation machinery. Both plasmids require an *E. coli* host containing the *pir*^+^ gene for replication (R6Kγ origin) and cannot replicate in the recipient. To test Tn*7* integration as a vehicle to engineer *N. aromaticivorans* and *R. sphaeroides*, we attempted this and related mating schemes (Fig. 1A through E).

We successfully used tri-parental mating to introduce Tn*7* into the *N. aromaticivorans* genome, albeit with variable efficiency (Fig. 1C; Fig. S1; Table S1). In these transposition experiments, the frequency of transconjugants obtained per viable cells ranged from 6 × 10^−5^ to 1 × 10^−7^, but the total number of transconjugants never exceeded 10,000/mL. However, subsequent mating experiments that included a conjugal helper strain [*E. coli* containing pEVS104, which encodes a functional copy of the RP4 conjugation machinery (37, 38)] in a quad-parental mating increased Tn*7* transposition efficiency when used with the same *N. aromaticivorans* recipient culture under otherwise identical conditions (Fig. 1C; *P* < 0.05, *t*-test). We confirmed that insertion of the Tn*7* transposon did not affect the growth of *N. aromaticivorans* in rich and minimal media (Fig. S2; Table S2). To gauge the stability of Tn*7* integrants, we sequentially cultured *N. aromaticivorans* cells containing a Tn*7* transposon for approximately 50 generations in the absence of selection. We found that 100% of cells maintained the Tn*7* selectable marker (Fig. 1E).

Tn*7* transposition into the *R. sphaeroides* genome required additional optimization. Our initial tri-parental mating scheme failed to recover transconjugants, although transconjugants could be obtained with addition of the conjugal helper in a quad-parental mating (Fig. 1D). We hypothesized that poor recovery of transconjugants was due to insufficient, transient expression of the Tn*7* transposase genes (*tnsABCD*). To test this hypothesis and increase Tn*7* transposition efficiencies, we cloned the transposase genes on a replicative plasmid in *R. sphaeroides* (pTNS^++^), producing a new recipient strain (Fig. 1B). When using pTNS^++^ as a recipient, transposition efficiencies significantly improved relative to wild type (WT) without the conjugal helper (Fig. 1D; *P* < 0.05, *t*-test). The pTNS^++^ plasmid is easily cured: 32% ± 5% of colonies had lost the plasmid after 24 h of non-selective growth. We found that *R. sphaeroides* strains with an insertion at *att*_Tn7_ grew similarly in rich medium but that there may be a slight growth defect from *att*_Tn*7*_ insertion in minimal medium (Fig. S2; Table S3). As a caveat, we note that *R. sphaeroides* underwent only a limited number of doublings when grown in minimal medium under our conditions; growth for more generations or under alternative conditions could reveal additional growth phenotypes of the *att*_Tn*7*_ insertion. As was the case with *N. aromaticivorans*, we also found that the Tn*7* insertion element remained stable when passaged in the absence of selection (Fig. 1E). From these experiments, we conclude that Tn*7* transposition is an effective and stable approach to integrate engineered DNA into *N. aromaticivorans* and *R. sphaeroides*.

### Synthetic, inducible promoters for *N. aromaticivorans* and *R. sphaeroides*

Synthetic, inducible promoters are valuable for strain engineering because they mitigate the effects of physiological regulation associated with native promoters and can be controlled in a temporal, reversible, and titratable manner. IPTG-inducible promoters are especially valuable for many studies because IPTG is metabolically inert and diffuses readily into most cells, and its interactions with Lac repressor (LacI) are extremely well characterized (39–42). These promoters are often designed such that LacI competes for a binding site on DNA with RNA polymerase (RNAP): addition of IPTG removes DNA binding by LacI, which allows RNAP binding. To identify synthetic, regulated promoters for *N. aromaticivorans* and *R. sphaeroides*, we assembled a set of 20 IPTG-inducible promoters (43) that vary in DNA sequences within the −35 and −10 elements (44), spacer length (45), presence or absence of a sequence upstream of the −35 element that interacts with the RNAP α-CTD known as the UP element (46), and *lac* operator sequence (47) (Tables S4 to S7). These promoters combined elements from the Anderson promoter library (48), UP sequences from Estrem et al. (49), and were based on a general understanding of *E. coli* σ^70^-DNA contacts (50–52).

**Fig 2:**
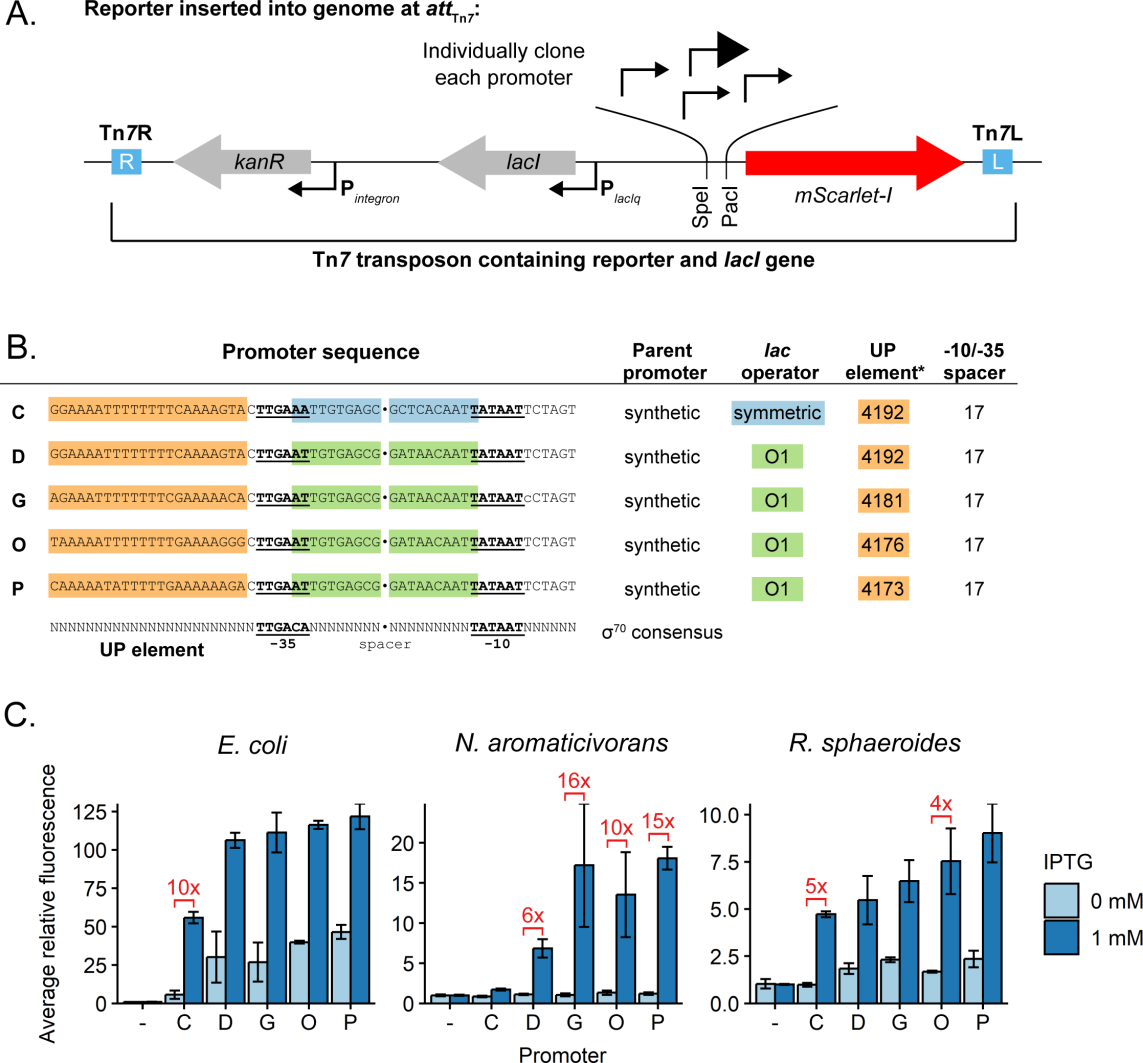
Selected high-activity promoters in *N. aromaticivorans* and *R. sphaeroides*. (**A**) Schematic of the Tn*7* transposon containing the test promoter construct, which is inserted into the genome in the *att*_Tn*7*_ site (sequences of individual promoters provided in Table S15). Promoters of interest were cloned upstream of the *mScarlet-I* gene using the indicated PacI and SpeI restriction sites. The *lacI* and *kanR* genes are transcribed from P*_lacIq_* and the class I integron promoter P_integron_, respectively. Blue boxes indicate Tn*7* transposon end sequences. (**B**) Sequences of five promoters that exhibit the most activity in both organisms. Promoter C includes a symmetric *lac* operator, while the four others include *lac* O1 operators. The most sequence differences between the high-activity promoters are bases in the UP element. *UP elements correspond to those in Estrem et al. (49). (**C**) Activity of each promoter in *E. coli*, *N. aromaticivorans*, and *R. sphaeroides*. A linear scale is used for the *Y* axis. Summary statistics can be found in Tables S2 to S5.

To screen this promoter library for activity, we built a Tn*7*-based fluorescent reporter system. Each of the 20 promoters was independently cloned upstream of the *mScarlet-I* gene (53) so that promoter activity could be measured by mScarlet-I fluorescence (Fig. 2A; Tables S4 to S7 and S15). We integrated these constructs separately into the *N. aromaticivorans* and *R. sphaeroides att*_Tn*7*_ sites and into the *E. coli att*_Tn*7*_ site as a comparator. Relative promoter activity was calculated by dividing the activity of each strain by the median activity of the least active of the 20 promoters in the respective host (Fig. S3). For our analysis, synthetic promoters with the same or lower activity than a control strain lacking a promoter were considered to have negligible activity.

We found several synthetic promoters that had activity above background and were IPTG inducible across the three organisms (Fig. 2B and C; Fig. S3). In general, promoters D, G, O, and P (P_D_, P_G_, P_O_, and P_P_) were active in both *N. aromaticivorans* and *R. sphaeroides*; these promoters contained near-consensus binding sites (−10 and −35) for *E. coli* σ^70^, underscoring the cross-species relevance of these sequences to activity. Promoters with the largest fold increases in activity upon induction differed across species. For instance, P_C_ showed the greatest fold induction in *E. coli* (~10-fold) and *R. sphaeroides* (~5-fold) but was largely inactive in *N. aromaticivorans* (~2-fold). Several promoters showed strong induction (10-fold or greater) in *N. aromaticivorans*, including P_G_, P_O_, and P_P_ (16-, 10-, and 15-fold, respectively). Induction effects were generally lower for *R. sphaeroides*, with P_C_ showing the best induction ratio (~5-fold). We also found that the most active *N. aromaticivorans* and *R. sphaeroides* promoters contained sequences with UP elements that had been previously isolated from a SELEX (systematic evolution of ligands by eXponential enrichment) screen for tight binding to *E. coli* RNA polymerase (49). This was somewhat surprising because such AT-rich, “canonical” UP elements are essentially absent from known alphaproteobacterial promoters due to their high genomic GC content (65% and 68.5% GC for *N. aromaticivorans* and *R. sphaeroides*, respectively) (54). In summary, we have developed synthetic, IPTG-inducible promoters for *N. aromaticivorans* and *R. sphaeroides* that can be used to control expression of engineered genes or pathways.

### Programmable gene knockdown in *N. aromaticivorans* and *R. sphaeroides* with CRISPRi

**Fig 3:**
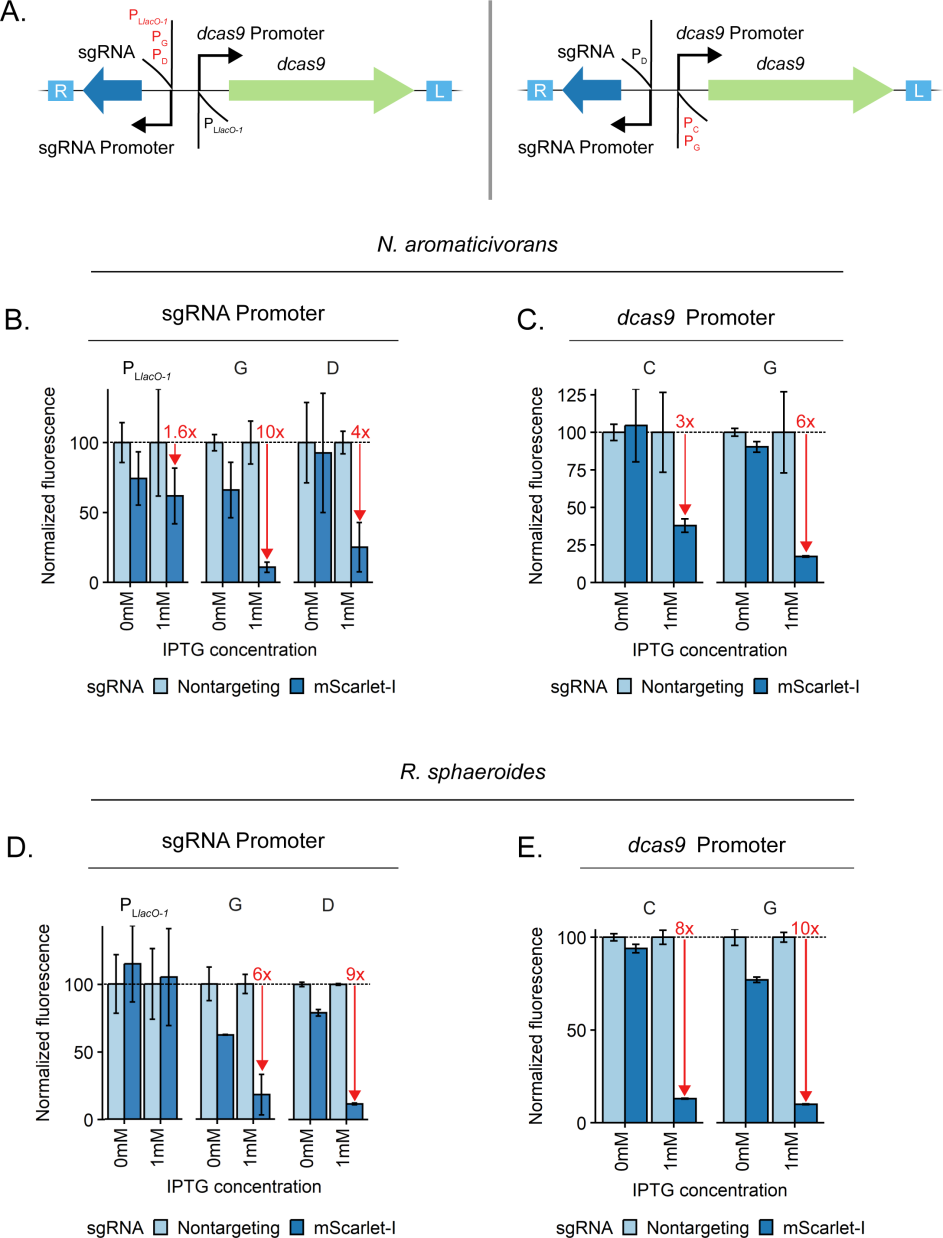
Optimization of Mobile-CRISPRi for use in *N. aromaticivorans* and *R. sphaeroides*. (**A**) Schematic of Mobile-CRISPRi system with varied sgRNA and dCas9 promoters. The *mScarlet-I* gene is also present in the Tn*7* transposon but is not shown here due to space constraints. (**B and D**) Use of different promoters to drive sgRNA expression to control *mScarlet-I* expression in *N. aromaticivorans* and *R. sphaeroides*, respectively. All constructs use *Spy-*dCas9 expressed from a P_L*lacO1*_ promoter. (**C and E**) Use of promoters C and G to drive *Spy*-dCas9 expression to control *mScarlet-I* expression in *N. aromaticivorans* and *R. sphaeroides,* respectively. Constructs use promoter D for sgRNA expression. Data normalization: for a given Mobile-CRISPRi vector, in the given condition, fluorescence per OD600 values for all independent replicates was normalized to the mean fluorescence per OD600 value for the strain encoding non-targeting control sgRNA, which was assigned to be a value of 100% expression. Summary statistics can be found in Tables S8 to S10.

A specific and significant reduction of gene expression at the transcriptional level can be achieved in many organisms using CRISPRi. CRISPRi utilizes a catalytically inactive Cas9 (dCas9) and gene-targeting sgRNA to physically block transcription, reducing gene transcript levels (55). To establish and test the utility of CRISPRi in *N. aromaticivorans* and *R. sphaeroides*, we began by testing the function of a previously developed Mobile-CRISPRi system which inserts in *att*_Tn*7*_ and has been shown to be effective in diverse bacteria (22, 36) (Fig. 3A).

Knockdown of fluorescent protein-encoding genes is an established approach to measure the efficacy of CRISPRi (23, 55–57). To that end, we used a constitutively expressed *mScarlet-I* reporter contained within the same Tn*7* construct as the CRISPRi components and either *mScarlet-I*-targeting sgRNAs or non-targeting controls (Fig. 3B through E; Tables S8 to S10). Our initial construct contained synthetic, IPTG-inducible P_L*lacO-1*_ promoters upstream of the sgRNA and *dcas9* genes (22). However, we found that this construct showed little to no knockdown in *N. aromaticivorans* and *R. sphaeroides* (Fig. 3B and D; Fig. S4). To optimize CRISPRi, we first swapped the existing sgRNA promoter for either P_D_ or P_G_, finding increased knockdown activity in both species (knockdown was normalized to non-targeting controls; Fig. 3B and D). Although the use of P_G_ for sgRNA expression showed the strongest CRISPRi knockdown in *N. aromaticivorans*, we elected to continue optimization with P_D_-sgRNA due to slightly less leaky knockdown in the absence of IPTG. We next substituted the *dcas9* promoter with either P_C_ or P_G_ and again tested knockdown activity, finding that P_G_-*dcas9* provided the most knockdown (Fig. 3C and E). That P_C_ or P_G_ provided negligible benefit over the initial P_L*lacO-1*_-*dcas9* construct suggests that sgRNA expression was likely limiting; however, we previously found that P_L*lacO-1*_ is an unstable promoter due to recombination between its two identical *lac* operator sites (58), so the use of P_G_ likely improves CRISPRi stability. We also avoided using the same promoter twice upstream of the sgRNA and *dcas9* genes to ensure construct stability.

**Fig 4:**
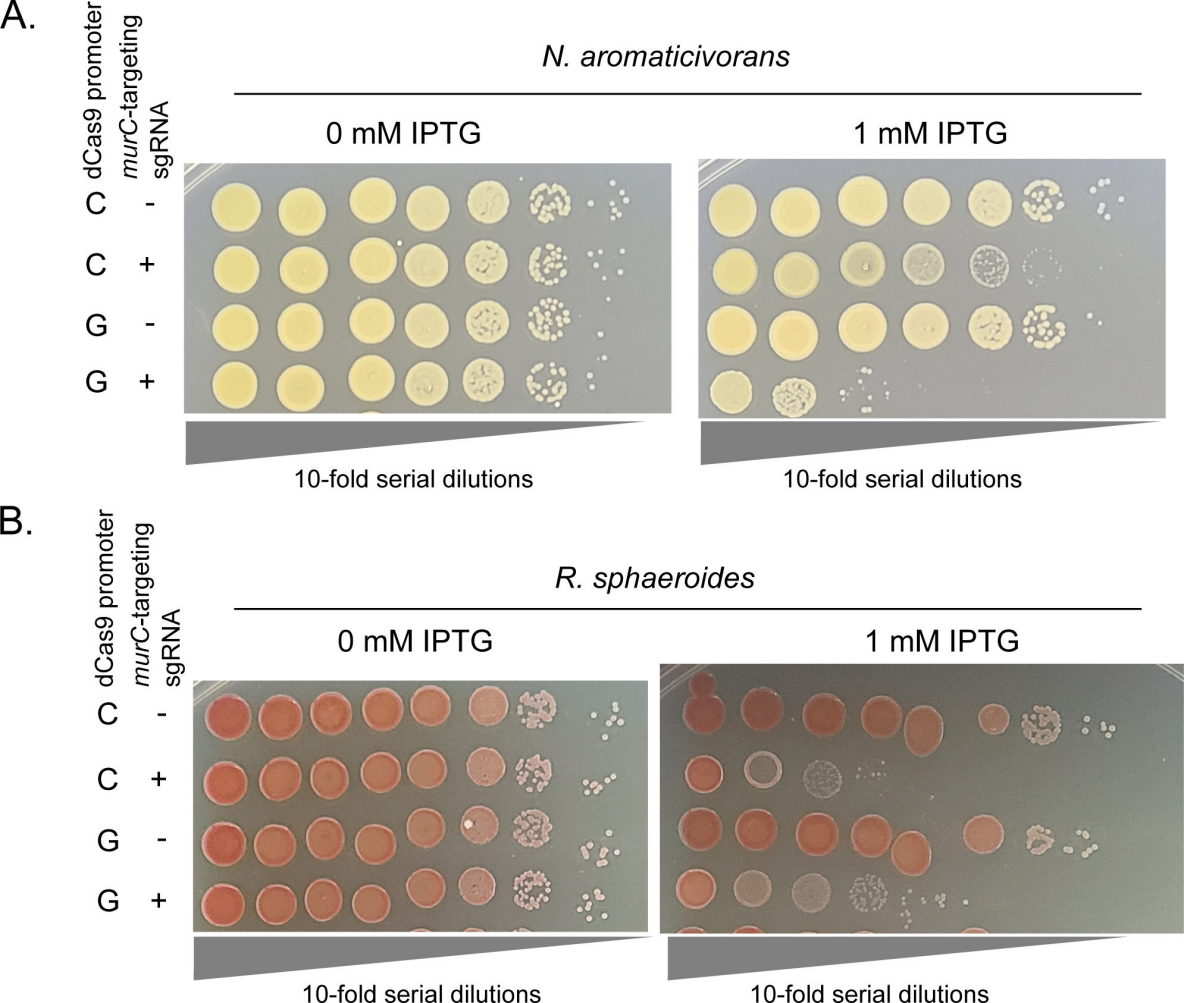
Controlled expression of an essential gene with Mobile-CRISPRi in *N. aromaticivorans* and *R. sphaeroides*. (**A**) Ten-fold serial dilutions of *N. aromaticivorans* with Mobile-CRISPRi constructs with a sgRNA targeting the essential gene *murC* or a non-targeting control sgRNA. *N. aromaticivorans* cells were normalized to an OD600 of 10 prior to serial dilution. Cells were grown on rich media (464a) in the presence or absence of 1-mM IPTG. (**B**) Ten-fold serial dilutions of *R. sphaeroides* with Mobile-CRISPRi constructs with an sgRNA targeting the essential gene *murC* or a non-targeting control sgRNA. *R. sphaeroides* cells were normalized to an OD600 of 10 prior to serial dilution. Cells were grown on rich media (LB [Lysogeny Broth]) in the presence or absence of 1-mM IPTG. Figure S5 shows additional biological replicates of each.

To test the ability of CRISPRi to inhibit expression of native genes in *N. aromaticivorans* and *R. sphaeroides*, we targeted the essential *murC* gene (11) that has been shown to be highly sensitive to inhibition in other Gram-negative bacteria (57). In these experiments, we tested the P_D_-driven sgRNA and P_C_- or P_G_-dependent dCas9 constructs that showed detectable knockdown when using *mScarlet-I* as a reporter. To measure the physiological effects of *murC* knockdown, we grew cells in the absence of induction prior to harvesting and spotting a 10-fold serial dilution series of each strain onto media including or lacking 1-mM IPTG (Fig. 4A and B; Fig. S5). In *N. aromaticivorans*, the P_G_-dependent dCas9 showed the best performance, yielding an ~10,000× reduction in viability consistent across all replicates, consistent with higher knockdown activity observed for P_G_ in the *mScarlet-I* reporter assay (Fig. 3C). In *R. sphaeroides*, the P_C_- and P_G_-dependent dCas9 constructs performed similarly, with ~1,000–10,000× reduction in viable colonies in both strains (Fig. 4B; Fig. S5). In both organisms, we observed no loss of viable colonies in the absence of IPTG, suggesting that expression of CRISPRi components was highly dependent on the addition of IPTG (Fig. 4; Fig. S5). Thus, we conclude that these Mobile-CRISPRi systems for *N. aromaticivorans* and *R. sphaeroides* are effective, tightly regulated, and capable of targeting endogenous essential genes in both organisms.

### Production and control of an engineered natural product in *N. aromaticivorans*

**Fig 5:**
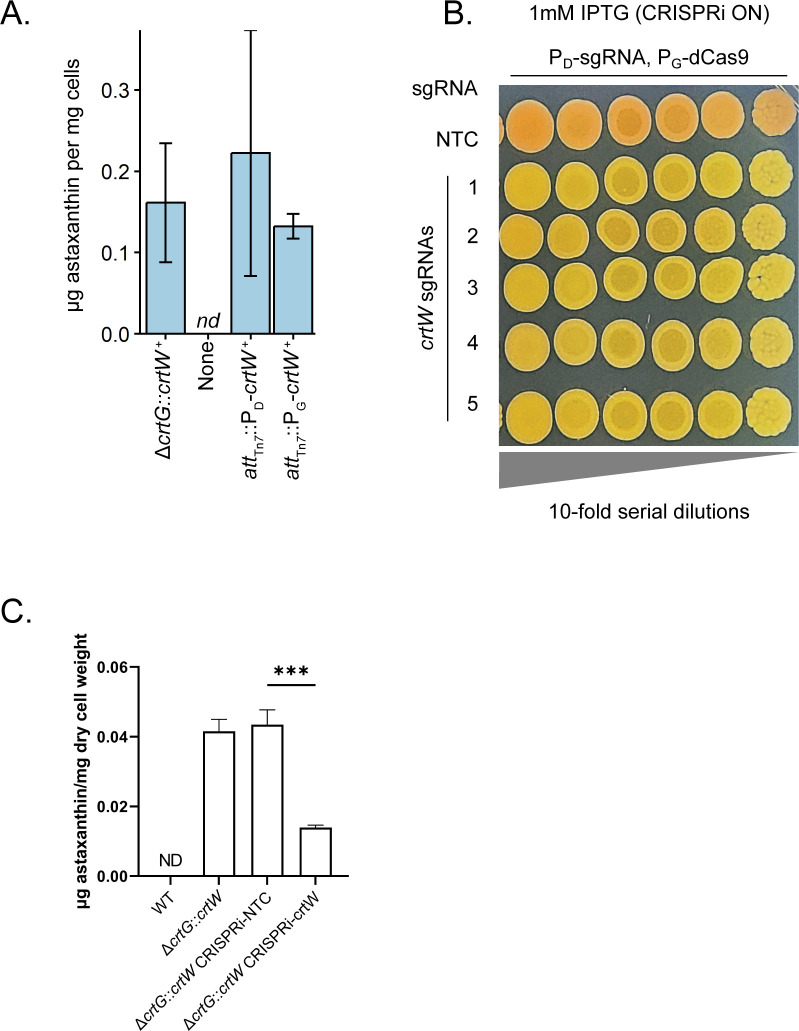
Expression control of the exogenous *crtW* gene in *N. aromaticivorans*. (**A**) Expression of *crtW* from the *att*_Tn*7*_ site in ∆*crtG N. aromaticivorans* produces comparable quantities of astaxanthin as a previously engineered expression strain (∆*crtG::crtW^+^*) (60). There is no difference in the amount of astaxanthin produced among all *crtW* strains; astaxanthin was not detectable in cells lacking this foreign gene (∆*crtG*, Tn*7* construct “none”). (**B**) Ten-fold serial dilutions of ∆*crtG::crtW^+^* with a Mobile-CRISPRi system at the *att*_Tn*7*_ site. Cells were plated on rich media (464a) supplemented with 1-mM IPTG. Summary statistics can be found in Table S11. (**C**) Reduction in astaxanthin production by CRISPRi. Mobile-CRISPRi with P_D_-sgRNA and P_G_-dCas9 was used to target *crtW* (CRISPRi-*crtW*) or contained an NTC sgRNA. Astaxanthin was quantified as described in Materials and Methods. Astaxanthin production was reduced by ~4-fold only when the sgRNA targeting *crtW* was present. ***Unpaired two-tailed student’s *t*-test, *P* < 0.001. nd or ND, not detected; NTC, non-targeting control.

*N. aromaticivorans* has potential as a bacterial chassis for the conversion of plant materials into low-volume, high-value bioproducts (2, 3, 12, 59). We recently demonstrated that the carotenoid astaxanthin can be produced by *N. aromaticivorans* by replacing the genomic copy of the native gene *crtG* with *crtW* from *Sphingomonas astaxanthinifaciens* (∆*crtG::crtW^+^*) (60). To demonstrate the utility of the Tn*7* insertion and inducible promoters described in this work to control the levels of an engineered pathway, we sought to build a strain of *N. aromaticivorans* capable of producing astaxanthin by expressing the exogenous *crtW* at the *att*_Tn*7*_ site (Fig. 5A; Table S11). We measured astaxanthin production in strains producing *crtW* from the *crtG* locus and from *att*_Tn*7*_ driven by promoters D and G. We found that all three strains accumulated comparable amounts of astaxanthin when grown in standard laboratory media (Fig. 5A).

As a proof of principle, we next used our Tn*7*/synthetic promoter/CRISPRi platform to control the expression of the foreign *crtW* gene (Fig. 5B). Using the ∆*crtG::crtW^+^* strain, we inserted Tn*7*-based CRISPRi constructs that carried either a non-targeting control guide or one targeting *crtW*. We also tested constructs in which P_C_ or P_G_ was used to control transcription of the *dcas9* gene. A reduction of CrtW expression by CRISPRi was apparent from colony images, as expression results in orange colonies due to astaxanthin accumulation (Fig. 5B). We also quantified astaxanthin production in CRISPRi knockdown strains (Fig. 5C), showing a decrease in astaxanthin abundance upon induction of CRISPRi with a sgRNA targeting *crtW* but not with a non-targeting guide. Taken together, we find that the combination of Tn*7*, select synthetic promoters, and CRISPRi can be used to effectively build and modulate gene expression of engineered constructs in *N. aromaticivorans*.

## DISCUSSION

There is a need for facile tools for genomic engineering and editing to dissect the lifestyle of Alphaproteobacteria and to remove barriers in efficiently constructing strains for production of bioproducts from abundant renewable raw materials including deconstructed lignocellulosic biomass. The suite of tools presented here for *N. aromaticivorans* and *R. sphaeroides* will advance our ability to engineer pathways and will accelerate strain optimization. We show that our platform of Tn*7*, synthetic promoters, and CRISPRi effectively delivers and modulates gene expression of native genes and engineered constructs in *N. aromaticivorans* and *R. sphaeroides*. These genetic tools can be applied to both gene function discovery and manipulating metabolic pathways, providing the means to identify engineering targets and further manipulate gene expression to obtain new knowledge about the biology of these microbes, possibly other Alphaproteobacteria, as well as other members of the bacterial phylogeny. We have summarized the most useful tools utilized in or constructed for this study in Table 1.

**TABLE 1:** Defined set of useful reagents described in this study

Reagent	Type^*a*^	Key features
pEVS104	Plasmid	Conjugal helper plasmid that increases conjugation efficiency, does not replicate in recipient cells (37)
pTNS^++^ (pJMP8045)	Plasmid	pBBR1 origin replicating vector expressing Tn*7* transposase genes, increases Tn*7* transconjugant recovery in *R. sphaeroides*
pJMP8602	Plasmid	Tn*7* plasmid containing a promoterless *mScarlet-I* gene for testing promoter activity
P_C_	Promoter	Synthetic, IPTG-inducible promoter in *R. sphaeroides* and *N. aromaticivorans*
P_D_	Promoter	Synthetic, IPTG-inducible promoter in *R. sphaeroides* and *N. aromaticivorans*
P_G_	Promoter	Synthetic, IPTG-ducible promoter in *R. sphaeroides* and *N. aromaticivorans*
P_O_	Promoter	Synthetic, IPTG-inducible promoter in *R. sphaeroides* and *N. aromaticivorans*
P_P_	Promoter	Synthetic, IPTG-inducible promoter in *R. sphaeroides* and *N. aromaticivorans*
pJMP8921	Plasmid	Mobile-CRISPRi plasmid containing mScarlet-I reporter, P_D_-sgRNA (BsaI/no spacer), and P_C_-dCas9 for testing system (no guide control)
pJMP8823	Plasmid	Mobile-CRISPRi plasmid containing mScarlet-I reporter, P_D_-mScar1 sgRNA, and P_C_-dCas9 for testing system (mScarlet-I targeting guide)
pJMP8930	Plasmid	Mobile-CRISPRi plasmid containing P_D_-sgRNA (BsaI/no spacer) and P_C_-dCas9 for cloning new sgRNA spacers
pJMP8923	Plasmid	Mobile-CRISPRi plasmid containing mScarlet-I reporter, P_D_-sgRNA (BsaI/no spacer), and P_G_-dCas9 for testing system (no guide control)
pJMP8835	Plasmid	Mobile-CRISPRi plasmid containing mScarlet-I reporter, P_D_-mScar1 sgRNA, and P_G_-dCas9 for testing system (mScarlet-I targeting guide)
pJMP8932	Plasmid	Mobile-CRISPRi plasmid containing P_D_-sgRNA (BsaI/no spacer) and P_G_-dCas9 for cloning new sgRNA spacers

^*a*^ See Fig. 2B and Table S15 for promoter sequences.

Our use and optimization of Tn*7* transposition in *N. aromaticivorans* and *R. sphaeroides* provide a rapid, straightforward approach for genomic integration of single genes or entire pathways, or gene knockdown libraries. For instance, we have previously used Tn*7* to construct a genome-scale CRISPRi library in *Z. mobilis* (61). With the efficiencies observed here, constructing pathway libraries on the order of thousands of permuted pathways should be feasible (e.g., thousands of different ribosome-binding sites with varying translation initiation rates); this will enable high-throughput screening of gene product and pathway function directly in *N. aromaticivorans* and *R. sphaeroides*. Although the cargo capacity of Tn*7* is unknown, the original Tn*7* element that contains ~14 kb of DNA (62) and Tn*7*-like elements associated with CRISPR systems (CAST) ranges in size from 22 to 120 kb (63). Therefore, we suggest that the size of the inserted DNA sequence is unlikely to be a limiting factor for Tn*7* integration. The Tn*7* attachment site can also be used for complementation analysis to verify that the function of a specific gene is central to an observed phenotype, allowing the investigator to avoid well-documented *cis*-acting affects, such as polarity, onto downstream genes as previously shown in other bacteria (64).

Our findings that Tn*7* transposition efficiency can be impacted by mating strategy and, presumably, transposase gene expression both sheds light on past difficulties in using this transposon in some species and opens additional avenues for increasing the recovery of transconjugants for library-scale studies. The inclusion of an additional *E. coli* donor strain containing a conjugal helper plasmid increased Tn*7* transconjugant recovery by an unknown mechanism. Although all *E. coli* strains present in the mating expressed conjugation machinery, it is possible that multicopy expression of conjugation pili drove recovery of additional transconjugants. We also observed similar behavior in *Acinetobacter baumannii* conjugations (65), indicating that the phenomenon could be broadly applicable. Expression of Tn*7* transposase genes in *R. sphaeroides* substantially increased recovery of transconjugants, suggesting that transposase gene expression was limiting.

Our identification of synthetic, inducible promoters for *N. aromaticivorans* and *R. sphaeroides* shows that some promoters built on the *E. coli* σ^70^ consensus can drive expression of native and foreign genes in Alphaproteobacteria, although not all promoters that were active in *E. coli* were also active in *N. aromaticivorans* and *R. sphaeroides*. It is possible that the reported inactivity of the *E. coli lac* promoter in *R. sphaeroides* (25) may be due to suboptimal −10 or −35 sequences, a wide spacer sequence (*lac* has an 18-bp spacer, rather than the more standard 17 bp), or some combination of elements. Interestingly, P_A_—a variant of the *lacUV5* promoter with an 18-bp spacer—shows higher relative activity in *N. aromaticivorans* than *R. sphaeroides*, suggesting there can be organism-specific differences in promoter sequence requirements even between closely related species. All of our promoters contained a “T” base at the −7 position of the −10 element. Therefore, we were unable to test if a substitution of this base would be tolerated in *N. aromaticivorans* and *R. sphaeroides* as recent work has shown for the ribosomal RNA promoter of *R. sphaeroides* (54). Retaining a T at the −7 position may still be advantageous in Alphaproteobacteria to avoid dependence on *trans*-acting factors such as the CarD transcription factor (32, 54, 66).

We predict that the use of CRISPRi in *N. aromaticivorans* and *R. sphaeroides* will provide a powerful new genetic tool to complement existing homology-mediated genome modification and Tn-seq in these and other Alphaproteobacteria (2, 3, 12). For example, CRISPRi will permit genome-scale interrogation of essential genes in *N. aromaticivorans* and *R. sphaeroides* as we have recently demonstrated in *Z. mobilis* (23). Also, Mismatch-CRISPRi—the use of sgRNAs that are deliberately modified to imperfectly match target DNA—will allow partial knockdowns of essential genes (57) that may be relevant for directing metabolic flux toward bioproducts. Combining CRISPRi functional genomics with metabolic models (67) may further accelerate rational engineering to increase bioproduct yields. We expect that these approaches will be useful in dissecting metabolic and regulatory networks in Alphaproteobacteria and in testing hypotheses to engineer strains for to improve bioproduct synthesis. Together, these advancements provide the foundation from which we can increase our understanding of microbial activities and to propel the bioeconomy.

## MATERIALS AND METHODS

### Strains and growth conditions

Table 1 lists strains used in this study. *E. coli* strains were grown in LB [10-g tryptone, 5-g yeast extract, 5-g sodium chloride (NaCl)/L; BD 240230] aerobically, either at 37°C in a flask with shaking at 250  rpm, in a culture tube on a roller drum, in a 96 deep-well plate with shaking at 900  rpm or at 30°C in a flask, or a culture tube with shaking at 250 rpm. *R. sphaeroides* strains were grown in Sistrom’s minimal media (SIS) consisting of K_2_HPO_4_, potassium phosphate anhydrous, 19.98 mM; ammonium sulfate, 3.78 mM; succinic acid, 33.87 mM; C_5_H_8_NO_4_K × H_2_O, glutamic acid (L), 0.68 mM; aspartic acid, 0.3 mM; NaCl, 8.56 mM; nitrilotriacetic acid, 1.05 mM; MgSO_4_ × 7H_2_O, magnesium sulfate, 1.22 mM; calcium chloride dihydrate, 0.23 mM; FeSO_4_ × 7H_2_O, ferrous sulfate, 7.19 µM; (NH4)_6_Mo_7_O_24_ × 4H_2_O, ammonium molybdate, 0.01 µM; EDTA disodium salt electrophoresis grade, 0.05 µM; ZnSO_4_ × 7H_2_O, zinc sulfate, 0.38 µM; FeSO_4_ × 7H_2_O, ferrous sulfate, 0.18 µM; MnSO_4_× 1H_2_O, manganese sulfate, 55 mM, 0.09 µM; CuSO_4_ × 5H_2_O, cupric sulfate, 0.02 µM; Co(NO_3_)2 × 6H_2_O, cobalt nitrate, 0.01 µM; boric acid, 0.02 µM; nicotinic acid, 8.12 µM; C_12_H_17_CIN_4_OS-HCl, thiamine hydrochloride, 1.48 µM; biotin (d), 0.04 µM (68) or LB at 30°C in a flask or a culture tube with shaking at 250 rpm. *N. aromaticivorans* strains were grown in 464 a (5 g tryptone, 5 g yeast extract, 1 g glucose per liter) (DSMZ) or SIS supplemented with 1% glucose.

For growth on plates, 15-g/L agar was added to the appropriate medium. When necessary, antibiotics were used at the following concentrations: *E. coli*, 100-µg/mL ampicillin (amp) or 30-µg/mL kanamycin (kan); *R. sphaeroides*, 25-µg/mL spectinomycin (spec), 25-µg/mL kan; *N. aromaticivorans*, 30-µg/mL kan, 15-µg/mL gentamycin. IPTG (1 mM) was added where indicated.

All strains were preserved in 15%–25% glycerol and frozen at −80°C.

### Conjugation and Tn*7* transposition

#### Growth of strains

##### Donor strains

The transposase donor strain sJMP2591 or sJMP11111, helper plasmid strain sJMP11115, and transposon donor strain (Table 1) were grown on LB agar supplemented with 300-µM diaminopimelic acid (DAP) and the appropriate antibiotic and grown 16–20 h at 37°C. Donor cultures were started from a single colony in 10-mL LB broth supplemented with DAP and amp and grown with shaking (250 rpm) at 30°C for 16–20 h. Alternately, cells were scraped from the primary streak after overnight growth and were resuspended in LB.

##### *E. coli* recipient

Strain sJMP3272 was streaked out on LB agar and grown overnight at 37°C. Recipient cultures were started from a single colony in 10-mL LB broth and grown with shaking (250 rpm) at 30°C for 16–20 h. Alternately, scrapes of each the donor and recipient strains were mixed on solid media, grown 2–8 h at 37°C, then streaked for single-colony isolates on selective media.

##### *R. sphaeroides* recipient

The mating strategy used depends on the recipient strain.

###### sJMP8063

*R. sphaeroides* containing the Tn*7* transposase-expressing plasmid pJMP8045 (strain sJMP8063) was streaked out on SIS media supplemented with 25-µg/mL spec and grown for 1 day at 30°C. A pre-culture from a scrape of cells from the primary streak was inoculated in 1 mL of SIS supplemented with 25-µg/mL spec and grown with shaking (250 rpm) at 30°C for 18–22 h. This 1-mL pre-culture was then added to 10-mL SIS supplemented with 25-µg/mL spec and grown with shaking (250 rpm) at 30°C for another 18–22 h prior to use in conjugation.

###### sJMP8005

*R. sphaeroides* (sJMP8005) WT recipient, *E. coli* transposase donor (sJMP11111 or sJMP2591), *E. coli* conjugal helper plasmid donor (sJMP11115), and the appropriate transposon donor (see Table 1) were used for conjugation.

### *N. aromaticivorans* recipient

Promoter-*mScarlet-I* and Fig. S4 CRISPRi construct *N. aromaticivorans* WT were streaked on 464a plates and incubated at 30°C for 2 days. A 10-mL 464a culture was inoculated from a single colony and grown for 16–20 h at 30°C in a 125-mL baffled flask with shaking at 250 rpm. Cells were harvested and resuspended to an OD600 of 3. One milliliter of cells was used in each mating, with 200 µL of each *E. coli* transposase and transposon donor, also normalized to an OD600 of 3. Cells were concentrated by centrifugation at 4,000 × *g*; the supernatant was decanted; and cells were resuspended in residual volume, spotted on plates, and allowed to mate overnight. Cells were then scraped into 1-mL 464a media, resuspended, serially diluted, and plated.

Figure 1 mating efficiency experiments, all other CRISPRi constructs and *crtW* strains of *N. aromaticivorans* WT (sJMP11093) were patched onto 464a plates directly from the freezer and incubated at 30°C for ~24 h. Cells were scraped into 464a media and normalized to an OD600 of 3. One milliliter of cells was combined with *E. coli* donor cells: 200-µL transposase donor at OD600 3, 400-µL transposon donor at OD600 3, and 400-µL conjugal helper at OD600 3. Cells were concentrated by centrifugation at 4,000 × *g*; the supernatant was decanted; and cells were resuspended in residual volume, spotted on plates, and allowed to mate for 4 h at 30°C. Mating spots were then scraped into 1-mL 464a media, resuspended, serially diluted, and plated.

### Cloning

#### Plasmid preparation

Plasmids were prepared from *E. coli* strains with one of the following kits: GeneJet Plasmid Miniprep Kit (K0503, Thermo Scientific), QIAprep Spin Miniprep Kit (27104, Qiagen), or the PureLink HiPure Plasmid Midiprep kit (K210005, Invitrogen).

#### Preparation of competent *E. coli* cells

Competent cells of strains sJMP3053 (*pir*^+^ cloning strain) and sJMP3257 (*pir*^+^, *dap*^−^ mating strain) were prepared as previously described (36). Briefly, *E. coli* cells were grown in LB (supplemented with DAP when appropriate) at 37°C to early log phase (OD600 ~0.3). Cells were immediately chilled on ice, harvested by centrifugation in a swinging-bucket rotor, then washed 3× in cold 5% glycerol. Aliquots of cells suspended in 15% glycerol were stored at −80°C for later use.

#### Transformation

*E. coli* cells were electroporated with the Bio-Rad Gene Pulser Xcell on the EC1 setting in 0.1-cm cuvettes. Cells were recovered in 800-µL LB media for 1 h at 37°C prior to plating on appropriate antibiotic, adding DAP when necessary.

#### Sequence validation

Sanger sequencing of select plasmid regions or select inserts was performed by Functional Biosciences (Madison, WI). Whole-plasmid long-read sequencing was performed by Plasmidsaurus (Eugene, OR) using Oxford nanopore technology.

#### pAlphabet-mScarlet-I constructs

Parent vector pJMP8602 was digested with restriction enzymes PacI (NEB) and SpeI (NEB). Complementary oligonucleotides with overhangs compatible with the PacI/SpeI-digested pJMP8602 sticky ends were annealed by combining equimolar amounts (2 µM each) in 1× Cutsmart buffer (NEB), heating to 95°C for 10 min, then allowing to gradually cool to room temperature. Annealed oligos were diluted 1:20, and 2 µL of annealed oligonucleotides was ligated into the digested pJMP8602 with T4 DNA ligase (NEB 0491) for either 2 h at room temperature or 16 h at 16°C. Two microliters of the ligation reaction was directly transformed into 50-µL electrocompetent sJMP3053 cells and plated on an appropriate selective medium. Clones were single colony purified prior to confirming plasmid construction and preservation at −80°C.

#### CRISPRi constructs

Parent Mobile-CRISPRi constructs were built as indicated in Table S13. sgRNA spacer sequences were cloned into the BsaI site of each relevant Mobile-CRISPRi vector as previously described (23, 36). Briefly, a 20-nucleotide sequence proximal to an NGG PAM sequence was identified, complementary to the gene of interest. Four-nucleotide ends were added to oligos encoding each strand, such that upon annealing, the paired strands will have a complementary overlap with the BsaI cut ends of the Mobile-CRISPRi vector. Sequences of oligonucleotides used to build CRISPRi constructs can be found in Table S14.

### Tn*7* insertion stability

#### N. aromaticivorans

Starter cultures were grown in 464a supplemented with kan overnight at 30°C with shaking at 250 rpm. One milliliter of culture was harvested by centrifugation; the supernatant was decanted and resuspended in 1-mL media lacking antibiotics. Cells were diluted 1:1,000 into fresh media and grown for 24 h. The process of diluting 1:1,000 and growing 24 h was repeated a total of five times for approximately 50 generations of growth in the absence of selection. Cells were serially diluted and plated on media lacking antibiotics, and 150 colonies per biological replicate were patched onto 464a and 464a + kan to determine the fraction that retained the Tn7 insertion.

#### R. sphaeroides

Samples were prepared as for *N. aromaticivorans* but using SIS media and an alternative dilution and timing process to account for the higher doubling time. The growth and dilution protocol was performed a total of six times, one of which was a 1:100 dilution, and the remainder of which were 1:1,000 dilutions. The total number of generations of growth in the absence of selection was approximately 50–55 generations.

### pTNS^++^ plasmid curing

A strain carrying pTNS^++^ was streaked out from the freezer onto rich media lacking antibiotics. Single colonies were inoculated in liquid media lacking antibiotics and grown 24 h, then serially diluted, and plated on media lacking antibiotics. Plates were incubated for 3 days to obtain colonies, which were patched onto selective and non-selective plates.

### Growth curves

Three biological replicates per genotype were grown to saturation in liquid media overnight, in the media in which the growth curve would be completed. Samples were diluted 1:1,000 (*N. aromaticivorans* complete and minimal media, *R. sphaeroides* complete media) or 1:100 (*R. sphaeroides* minimal media) into a total volume of 200 µL in a clear, flat-bottomed 96-well plate (Corning). IPTG was used at 1 mM. Growth curves were performed in a Tecan Sunrise or Tecan Infinite 200 Pro Mplex at 30°C, measuring OD600 every 15 min. Individual growth curves are shown in Fig. S6.

Data from the growth curves were analyzed in R with the growthcurver package (69).

### Fluorescence assays

Starter cultures were prepared either by growing in liquid medium overnight from single-colony isolates or by patching single-colony isolates onto agar plates, and the resulting growth was scraped into liquid and OD600-normalized the next day.

Starter cultures were diluted and inoculated into 96-deep well plates containing the appropriate media for the species analyzed, with or without 1-mM IPTG. Plates were incubated at 30°C on a plate shaker (Benchmark Orbi-shaker MP) set to 1,000 rpm until cells had grown to saturation. Two hundred microliters of each culture was transferred to a black, clear-bottom plate for analysis in a Tecan Mplex Infinite platereader.

### Spot plate assays

Triplicate single-colony isolates of *N. aromaticivorans* or *R. sphaeroides* were patched onto appropriate media lacking inducer and were grown overnight at 30°C. Cells were scraped off the plate and resuspended in 1-mL appropriate media (464a or LB) and normalized to an OD600 of 10. Ten-fold serial dilutions of each strain were prepared in 96-well plates, and 5 µL of each was spotted onto 15-cm petri plates containing the appropriate media either with or without 1-mM IPTG. Plates were grown at 30°C and imaged over 2–5 days in a lightbox with a Samsung Galaxy S20+ phone camera.

### Quantification of astaxanthin

Astaxanthin levels were quantified as previously described in Hall et al. (60).

#### Cell growth

Starter cultures were made by inoculating single colonies of *N. aromaticivorans* isolates in 2-mL 464a and growing for approximately 15 h at 30°C (not to saturation). Samples were diluted 1:50 into a total volume of 25-mL 464a, and 1-mM IPTG was added to appropriate samples. Cells were grown 9 h at 30°C with shaking at 250 rpm. Two 10-mL aliquots were harvested from each culture by centrifugation and processed as follows: (i) the supernatant was decanted and the pellet was allowed to dry in chemical fume hood to calculate dry cell weight; and (ii) the supernatant was decanted and the pellets were flash frozen in liquid nitrogen and stored at −80°C until preparation of lipophilic extracts.

#### For the CRISPRi crtW knockdown experiment

Starter cultures were made by inoculating single colonies of *N. aromaticivorans* isolates in 3-mL 464a and grown for 48 h at 30°C while shaking at 250 rpm. Samples were diluted 1:50 into a total volume of 5-mL 464a, and 1-mM IPTG was added to all samples. After 24 h, cells were again diluted 1:50 into 25-mL fresh 464a with 1-mM IPTG. After an additional 24 h, two 10-mL aliquots were harvested from each culture by centrifugation and processed as follows: (i) the supernatant was decanted and the pellet was allowed to dry in chemical fume hood to calculate dry cell weight, and (ii) the supernatant was decanted and the pellets were flash frozen in liquid nitrogen and stored at −80°C until preparation of lipophilic extracts.

#### Preparation of lipophilic extracts

Cell pellets were resuspended in 200-µL water, then transferred into a 15-mL centrifuge tube. Four milliliters extraction solvent (7:2 acetone:methanol solution) was added, and the samples were mixed by pipetting. The tube was centrifuged (10,000 × *g* for 20 min), then the supernatant was transferred to a new 15-mL tube. The pelleted cells were extracted a second time, adding 100-µL water for resuspension followed by 4-mL extraction solvent. After centrifugation, the supernatants from both extractions were combined. The combined supernatants were partially dried under a stream of N2 (to a final volume of ~1 to 3 mL) to concentrate materials before analysis by high-performance liquid chromatography (HPLC).

#### HPLC identification and quantification of astaxanthin

Acetone:methanol lipophilic extracts were analyzed via reverse-phase HPLC using a Kinetex 2.6-µm PS C18 100 Å (150 × 2.1 mm) column (Phenomenex, Torrance, CA) attached to a Shimadzu Nexera XR HPLC system. The mobile phase was a binary gradient of Solvent A (70% acetonitrile/30% water) and Solvent B (70% acetonitrile/30% isopropanol) flowing at 0.45 mL/min for 30 min. Absorbance was measured between 200 and 600 nm using a Shimadzu SPD-M20A photodiode array detector. An astaxanthin commercial standard (Sigma-Aldrich) was used to identify and quantify astaxanthin in the extracts. Astaxanthin was eluted at a retention time of 12.6 min, corresponding to approximately 30% Solvent B.

## Data Availability

Selected plasmids and their sequences are available from Addgene (Addgene identification numbers 220900–220906).

## SUPPLEMENTAL MATERIAL

Supplemental figures aem.00348-24-s0001.pdf Figures S1 to S5.

Supplemental tables aem.00348-24-s0002.xlsx Tables S1 to S16.

ASM does not own the copyrights to Supplemental Material that may be linked to, or accessed through, an article. The authors have granted ASM a non-exclusive, world-wide license to publish the Supplemental Material files. Please contact the corresponding author directly for reuse.
