# Supplementary material for: Tools for genetic engineering and gene expression control in Novosphingobium aromaticivorans and Rhodobacter sphaeroides
Source: Appl Environ Microbiol. 2024 Sep 26;90(10):e00348-24. doi: 10.1128/aem.00348-24 (PMC11497788; doi:10.1128/aem.00348-24)
Supplement: Supplemental figures — Figures S1 to S5. [file aem.00348-24-s0001.pdf]

Figure S1

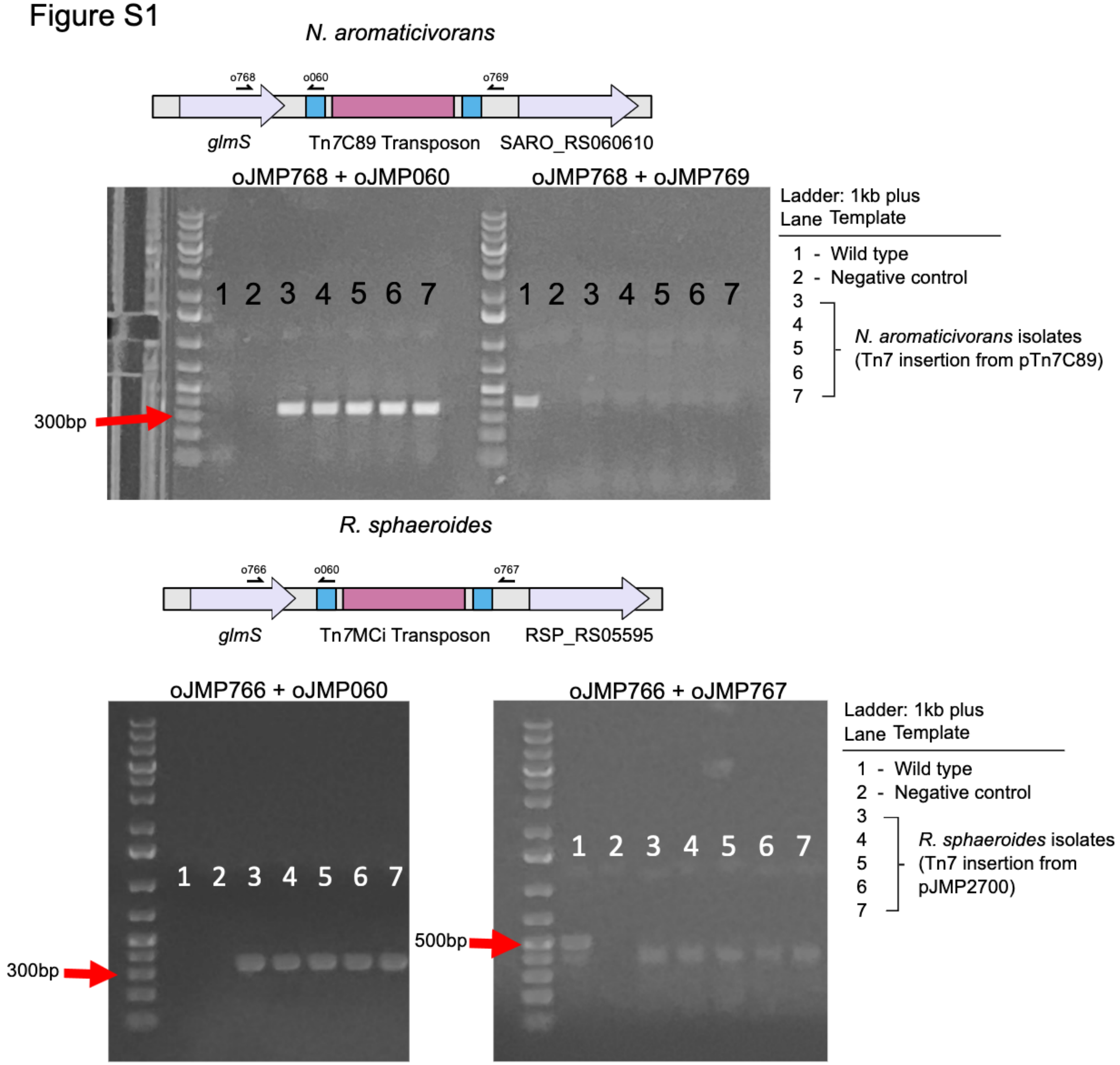

N. aromaticivorans
o768
o060
o769
glmS
Tn7C89 Transposon
SARO_RS060610
oJMP768 + oJMP060
oJMP768 + oJMP769
1 2 3 4 5 6 7
1 2 3 4 5 6 7
300bp
Ladder: 1kb plus
Lane Template
1 - Wild type
2 - Negative control
3
4
5
6
7
N. aromaticivorans isolates
(Tn7 insertion from pTn7C89)
R. sphaeroides
o766
o060
o767
glmS
Tn7MCi Transposon
RSP_RS05595
oJMP766 + oJMP060
oJMP766 + oJMP767
1 2 3 4 5 6 7
1 2 3 4 5 6 7
300bp
500bp
Ladder: 1kb plus
Lane Template
1 - Wild type
2 - Negative control
3
4
5
6
7
R. sphaeroides isolates
(Tn7 insertion from
pJMP2700)

Figure S2

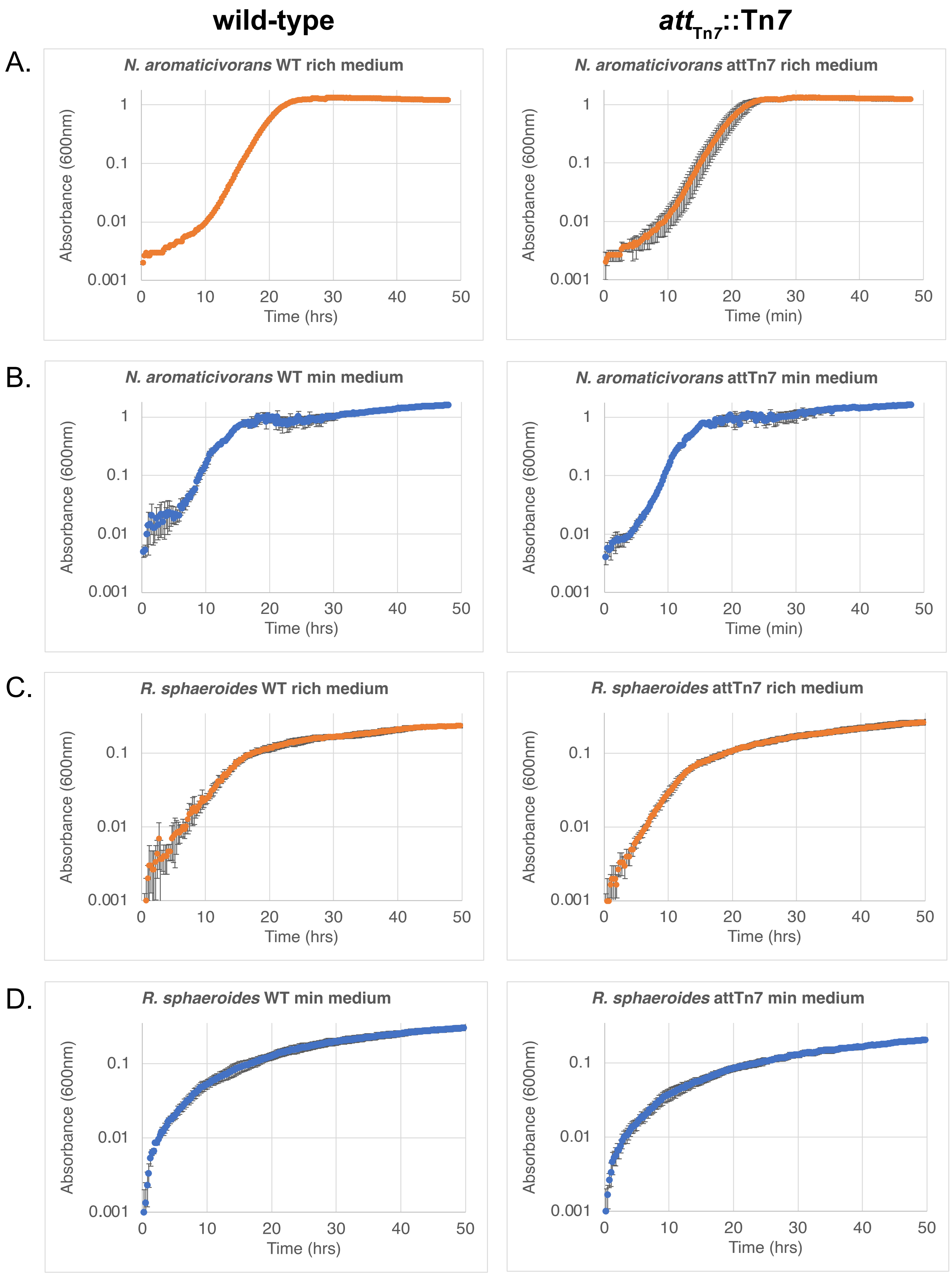

wild-type
att_Tn7::Tn7
A.
N. aromaticivorans WT rich medium
N. aromaticivorans attTn7 rich medium
B.
N. aromaticivorans WT min medium
N. aromaticivorans attTn7 min medium
C.
R. sphaeroides WT rich medium
R. sphaeroides attTn7 rich medium
D.
R. sphaeroides WT min medium
R. sphaeroides attTn7 min medium
Absorbance (600nm)
1
0.1
0.01
0.001
0
10
20
30
40
50
Time (hrs)
Time (min)

Figure S3

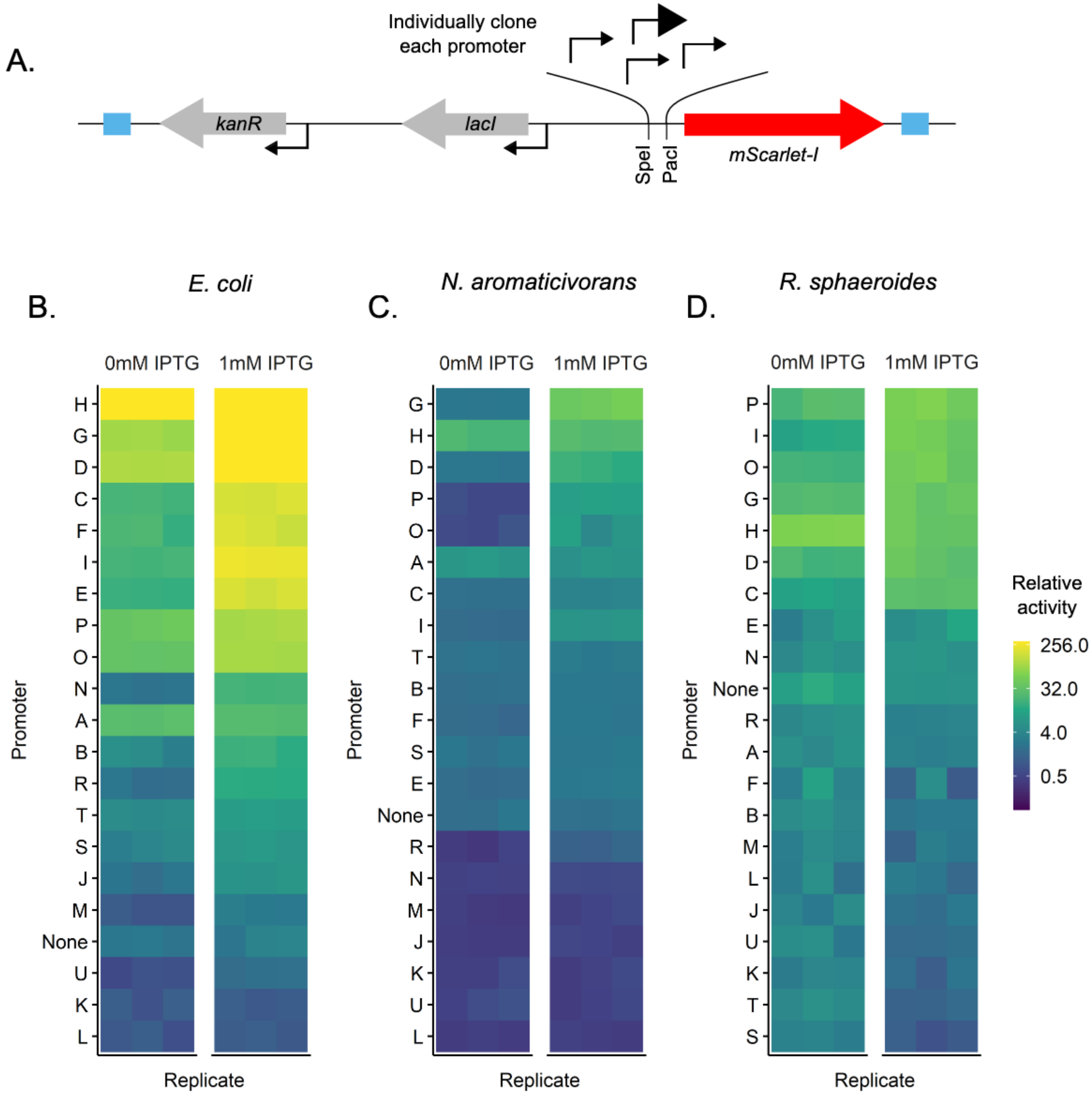
A.
Individually clone
each promoter
kanR
lacI
SpeI
PacI
mScarlet-I
B.
E. coli
C.
N. aromaticivorans
D.
R. sphaeroides
0mM IPTG
1mM IPTG
Promoter
Replicate
H G D C F I E P O N A B R T S J M None U K L
G H D P O A C I T B F S E None R N M J K U L
P I O G H D C E N None R A F B M L J U K T S
Relative activity
256.0
32.0
4.0
0.5

Figure S4

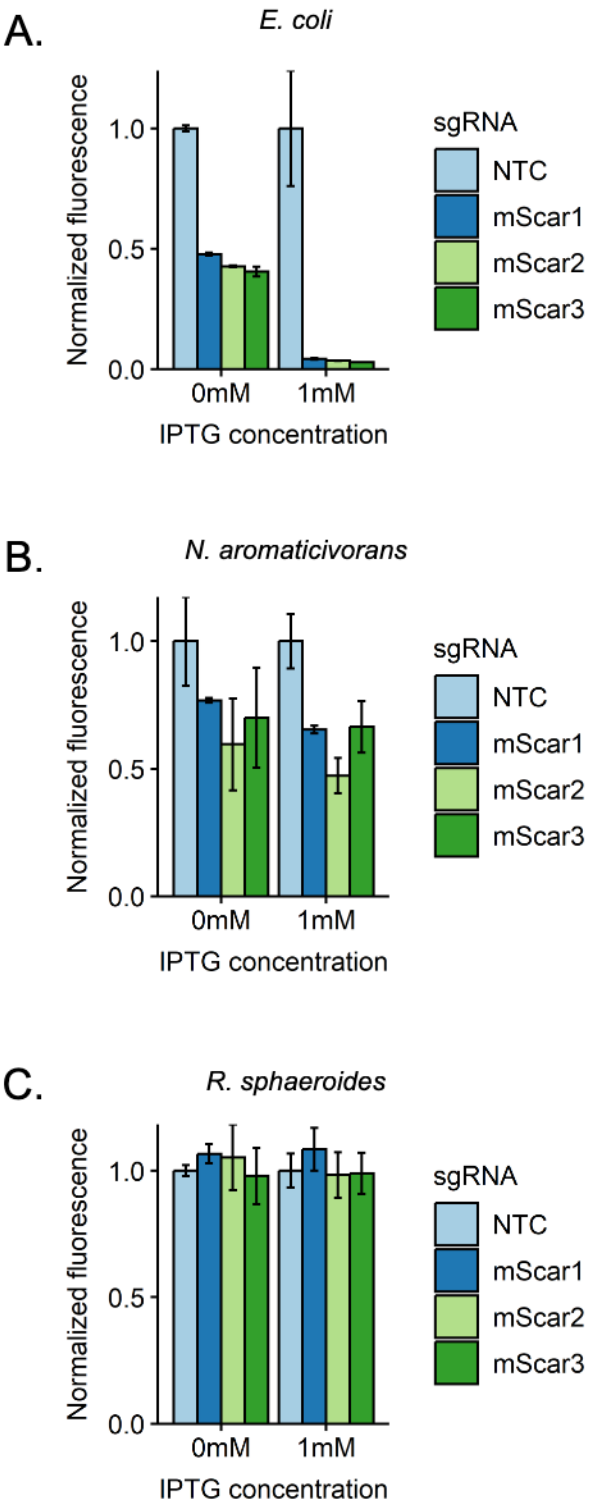

A.
E. coli
Normalized fluorescence
1.0
0.5
0.0
0mM
1mM
IPTG concentration
sgRNA
NTC
mScar1
mScar2
mScar3
B.
N. aromaticivorans
Normalized fluorescence
1.0
0.5
0.0
0mM
1mM
IPTG concentration
sgRNA
NTC
mScar1
mScar2
mScar3
C.
R. sphaeroides
Normalized fluorescence
1.0
0.5
0.0
0mM
1mM
IPTG concentration
sgRNA
NTC
mScar1
mScar2
mScar3

Figure S5

A.

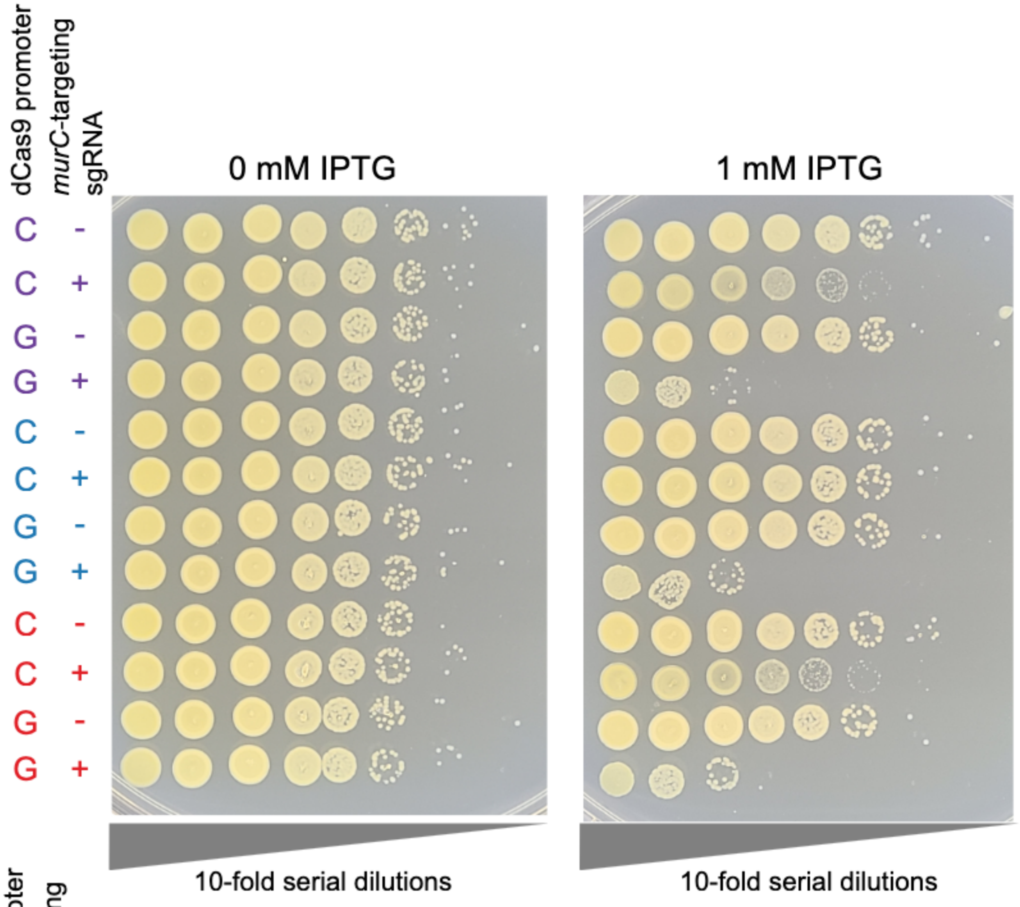
dCas9 promoter
murC-targeting sgRNA
0 mM IPTG
1 mM IPTG
C -
C +
G -
G +
C -
C +
G -
G +
C -
C +
G -
G +
10-fold serial dilutions
10-fold serial dilutions

B.

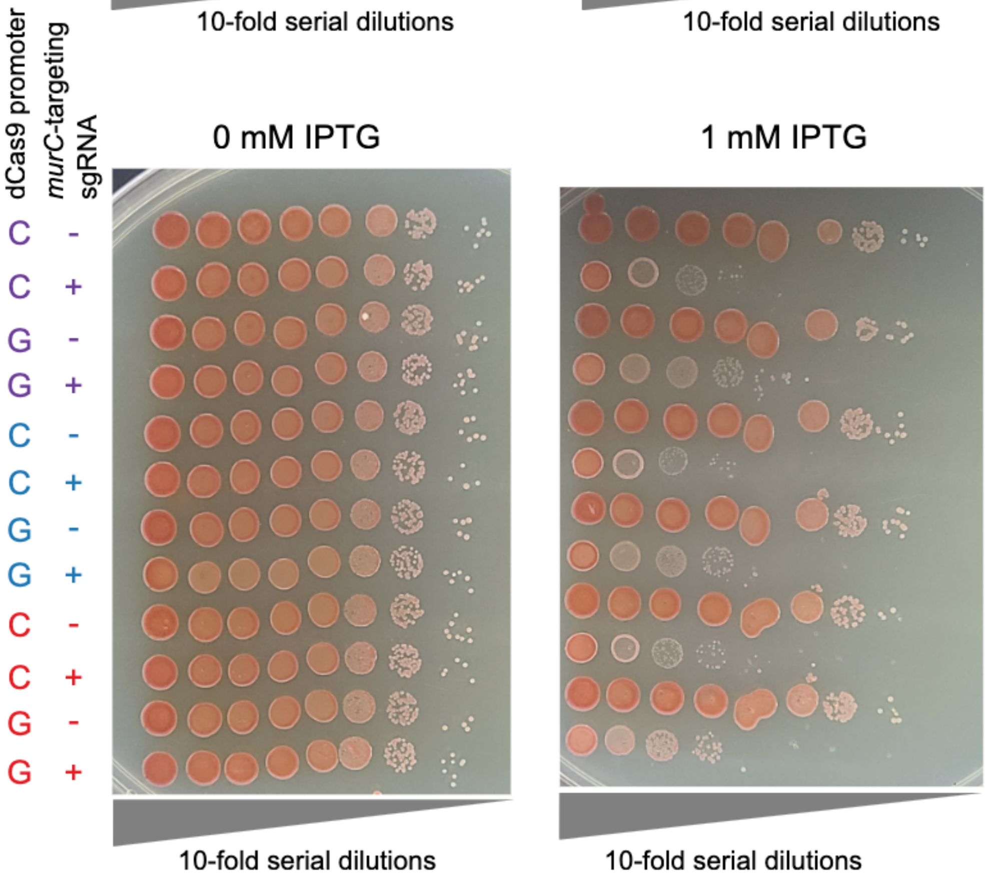
dCas9 promoter
murC-targeting sgRNA
0 mM IPTG
1 mM IPTG
C -
C +
G -
G +
C -
C +
G -
G +
C -
C +
G -
G +
10-fold serial dilutions
10-fold serial dilutions

SUPPLEMENTARY FIGURE LEGENDS

Figure S1: Confirmation of *att*::$_{Tn7}$ integration. A: Schematic of *glmS* genetic region with and without *att*::$_{Tn7}$ integration and locations of validation primers. This diagram is not to scale. B: *N. aromaticivorans* clones. C: *R. sphaeroides* clones. The negative control is a no template control.

Figure S2. Individual growth curves of *N. aromaticivorans* and *R. sphaeroides*. *N. aromaticivorans* growth curves in (A) rich medium (464a) with or without an insertion at *att*$_{Tn7}$, or (B) minimal medium (SIS + glucose) with or without an insertion at *att*$_{Tn7}$ and *R. sphaeroides* growth curves in (C) rich medium (LB) with or without an insertion at *att*$_{Tn7}$, or (D) minimal medium (SIS + glucose) with or without an insertion at *att*$_{Tn7}$. *R. sphaeroides* strains do not contain pTNS^{++} in these growth curves, as these strains were not generated using pTNS++ at any step during construction.

Figure S3: IPTG-inducible promoters expressing an *mScarlet-I* fluorescent reporter gene. A: Schematic of the Tn*7* transposon containing the test promoter construct (sequences of individual promoters provided in Table 4). Promoters of interest were cloned upstream of the *mScarlet-I* gene using the indicated PacI and SpeI restriction sites. B-D: Relative promoter activity in each *E. coli*, *N. aromaticivorans*, and *R. sphaeroides* in the presence and absence of IPTG. Relative activity was calculated by dividing each fluorescence-per-cell measurement by the median value of the least active promoter. Data are shown on a log2 scale. Promoter activity values and summary statistics can be found in Tables S4-S7.

Figure S4: CRISPRi knockdown of *mScarlet-I* with initial Mobile-CRISPRi constructs. Constructs with both sgRNA and dCas9 under the control of P_{LlacO1} were tested with three different targeting sgRNAs in *E. coli* (A), *N. aromaticivorans* (B), and *R. sphaeroides* (C). NTC=Non-targeting control strain.

Figure S5: Expanded data for *murC* CRISPRi in *N. aromaticivorans* and *R. sphaeroides*. A: 10-fold serial dilutions of *N. aromaticivorans* with Mobile-CRISPRi constructs with a sgRNA targeting the essential gene *murC* or a non-targeting control sgRNA. *N. aromaticivorans* cells were normalized to an OD600 of 10 prior to serial dilution. Cells were grown on rich media (464a) in the presence or absence of 1mM IPTG. B: 10-fold serial dilutions of *R. sphaeroides* with Mobile-CRISPRi constructs with a sgRNA targeting the essential gene *murC* or a non-targeting control sgRNA. *R. sphaeroides* cells were normalized to an OD600 of 10 prior to serial dilution. Cells were grown on rich media (LB) in the presence or absence of 1mM IPTG. The assay contains three biological replicates and samples presented in the main text are indicated in purple, and other replicates are labeled in blue and red.
